# More Accurate Transcript Assembly via Parameter Advising

**DOI:** 10.1089/cmb.2019.0286

**Published:** 2020-08-04

**Authors:** Dan Deblasio, Kwanho Kim, Carl Kingsford

**Affiliations:** ^1^ Computational Biology Department, Carnegie Mellon University, Pittsburgh, Pennsylvania, USA.; ^2^ Current affiliation: Department of Computer Science, The University of Texas at EI Paso, EI Paso, Texas, USA.; ^3^ Current affiliation: Broad Institute of MIT and Harvard, Cambridge, Massachusetts, USA.

**Keywords:** automated bioinformatics, genomics, parameter advising, transcript assembly

## Abstract

**Computational tools used for genomic analyses are becoming more accurate but also increasingly sophisticated and complex. This introduces a new problem in that these pieces of software have a large number of tunable parameters that often have a large influence on the results that are reported. We quantify the impact of parameter choice on transcript assembly and take some first steps toward generating a truly automated genomic analysis pipeline by developing a method for automatically choosing input-specific parameter values for reference-based transcript assembly using the Scallop tool. By choosing parameter values for each input, the area under the receiver operator characteristic curve (AUC) when comparing assembled transcripts to a reference transcriptome is increased by an average of 28.9% over using only the default parameter choices on 1595 RNA-Seq samples in the Sequence Read Archive. This approach is general, and when applied to StringTie, it increases the AUC by an average of 13.1% on a set of 65 RNA-Seq experiments from ENCODE. Parameter advisors for both Scallop and StringTie are available on Github**.

## 1. Introduction

**FIG. 1.**
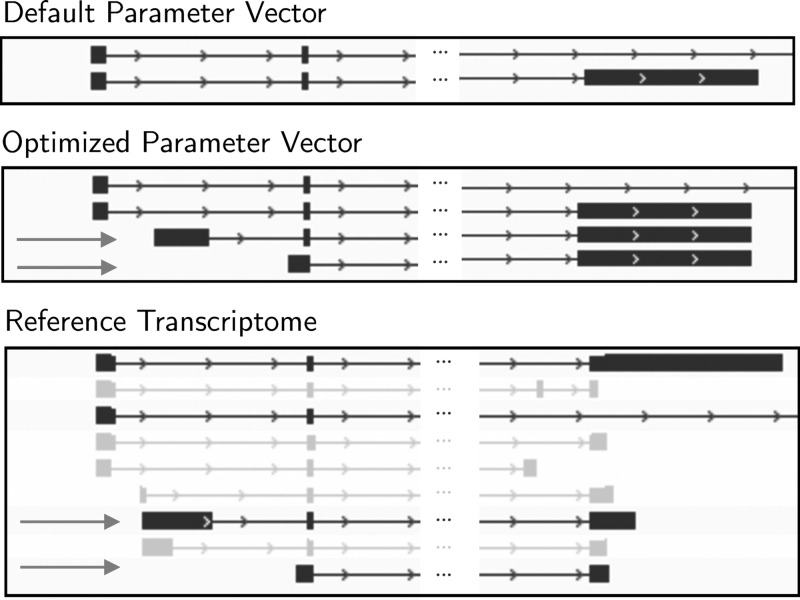
Influence of parameter choice on the produced transcriptome. The three sections of transcript assemblies are those assembled using Scallop's default parameter vector, an optimized parameter vector, and the reference transcriptome for positions 30231125–30260786 on Chromosome 2 in SRR543291/HISAT. The dashed arrows highlight the two transcripts from the reference that are not recovered using the default parameter vector.

As the field of computational biology has matured, there has been a significant increase in the amount of data that need to be processed and a corresponding increase in the reliance of users without computational expertise on the highly complicated programs that perform the analyses. At the same time, the number and sophistication of such tools have also increased. While the accuracy of such applications is constantly improving, a new problem has emerged: the sometimes overwhelming number of tunable parameters that each of these pieces of software brings with it. Changing an application's parameter settings can have a large influence on the quality of the results produced (Fig. 1). When incorrect or nonideal parameter choices are used, poorer results may be obtained or false conclusions may be reported.

The default parameter choices that most users rely on for these programs are typically optimized by the algorithm designer to maximize performance on the average case. This can be a problem since the most interesting experiments are often not “average.”

Manually tuning the parameter settings of an application often produces more accurate results, but it is very time-consuming. The tuning process can be accelerated for users with domain and/or algorithmic knowledge, as these experts can make more informed decisions about the correct direction to proceed when altering parameter values. However, tuning the parameter choices to increase accuracy for one input does not imply that the results will be improved for all inputs. This means that, for optimum performance, tuning must be repeated for each new piece of data. In the case of high-throughput genomic analysis, this manual procedure is infeasible. For these applications, without some sort of automatic parameter choice system, the defaults must be used.

To address the automated parameter choice problem for multiple sequence alignment (MSA), DeBlasio and Kececioglu (2017b) have defined a framework to automatically select the parameter values for an input. This process, called “parameter advising,” has been shown to greatly increase the accuracy of MSA without sacrificing wall-clock running time in most cases, and it can readily be applied to new domains.

**FIG. 2.**
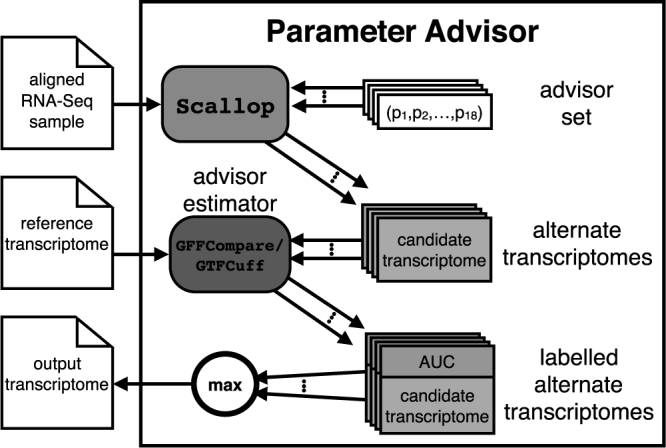
The Scallop parameter advisor. The advisor's input mirrors that of Scallop (an RNA-Seq sample that has been aligned to the reference genome). A set of candidate assemblies is created by running Scallop on each parameter vector in the advisor set. The advisor then returns the assembly with the highest AUC value, obtained by running GFFCompare and GTFCuff. AUC, area under the receiver operator characteristic curve.

In this work, we improve the quality of reference-based transcriptome assembly by extending parameter advising. Our new parameter advisor for transcript assembly is depicted in Figure 2. Details are provided in the next section. Transcriptome assembly takes an RNA-Seq sample and a reference genome as input and reconstructs the set of transcripts that are present. Common tools for reference-based transcript assembly include Scallop (Shao and Kingsford, 2017), TransComb (Liu et al., 2016), StringTie (Pertea et al., 2015), and Cufflinks (Trapnell et al., 2010). Reference-based assemblers first align reads to the reference genome using a tool such as HISAT (Kim et al., 2015), STAR (Dobin et al., 2013), TopHat2 (Kim et al., 2013), or SpliceMap (Au et al., 2010). Using the read splice locations (the positions where a read maps to non-neighboring locations on a genome), the assembler constructs the exons and splice-junctions of each transcript. The produced transcriptome consists of a combination of transcripts that can be mapped to ones we already know and transcripts that are unique to the sample that was assembled. These transcriptomes are used to perform analyses such as expression quantification (Bray et al., 2016; Patro et al., 2017) and differential expression (Love et al., 2014; Frazee et al., 2015).

For transcriptome assembly, the quality of a program's output is commonly measured by comparing it with a large set of reference transcripts, known as the reference transcriptome. We use the same measure for selecting parameter choices for a given input.

### 1.1. Contributions

We show for the first time that advising sets of parameter vectors can be constructed for tools with large numbers of tunable parameters. Second, we take some of the first steps toward producing a fully automated transcript analysis pipeline by automating sample-specific parameter selection for multiple applications. Third, we show that the commonly used reference-transcriptome-based quality is a better measure to use for parameter optimization in reference-based transcript assembly than other existing de novo metrics.

We show that by applying the parameter advising framework, we can increase the quality of the transcriptomes produced using the Scallop assembly tool. Using this approach, the area under the receiver operator characteristic curve (AUC, detailed in the next section) shows a median increase of 8.7% over using only the default parameter vector on a set of 10 RNA-Seq experiments contained in the ENCODE database that are commonly used for benchmarking. In a high-throughput pipeline, the median improvement is even larger: 28.9% higher AUC than the default parameter vector on over 1500 samples from the Sequence Read Archive.

We also confirm that this method can increase AUC for other programs by applying it to StringTie, another popular reference-based transcript assembler. For a set of 65 examples from the ENCODE database, we are able to increase StringTie's AUC by 13.1% over using only the default parameter vectors.

A previous version of work was presented at the 2019 Workshop for Computational Biology at the International Conference on Machine Learning (DeBlasio et al., 2019).

## 2. Developing a Parameter Advisor for Transcript Assembly

A parameter advisor has two components: (1) a set of parameter vectors—assignments of a value to each of the tunable parameters for the application—called an “advisor set”; and (2) an assessment criteria—a method to rank the quality of multiple solutions—called an “advisor estimator.” The advisor selects the appropriate parameter vector by first running the application on the input using each parameter vector in the set, and selecting the parameter vector that produces the best result according to the accuracy estimator. The instantiations of the application being tuned are independent processes that can be executed in parallel. Assuming that the number of processors available is at least the number of parameter vectors in the advisor set, the only additional wall time is the assessment of the results using the accuracy estimator (which can also be performed in parallel) and the comparison of these values, both of which are negligible compared with the running time of the application in most cases.

Parameter advising is an example of *a posteriori* parameter selection—it examines an application's output to select a parameter setting. In contrast, separate work has been done in other fields on *a priori* selection, where the parameters are chosen in advance by looking at the raw input alone, or a subsample of the input along with its result. This includes work such as Param-Medic (May et al., 2017) for choosing mass-spectrometry database search parameters, KmerGenie (Chikhi and Medvedev, 2013) for finding appropriate *k*-mer sizes for genomic assembly, and GRAPE (Majoros and Salzberg, 2004), which finds model parameters for gene finding. There is also work on this problem outside of computational biology such as ParamILS (Hutter et al., 2009), which finds optimal settings for the CPLEX computational optimization tool, SATZilla (Xu et al., 2008) for choosing from a collection of SAT solvers, as well as many tools developed for tuning hyperparameters in machine learning such as TPOT (Olson et al., 2016), which uses genetic algorithms, and Spearmint (Snoek et al., 2012), which uses Bayesian optimization. *A priori* prediction is necessary in cases when it is not feasible to apply multiple configurations, but more information is available when performing *a posteriori* assessment since the full final solution can be examined.

### 2.1. Advisor estimator

For transcriptome assembly, a natural choice for the estimator is the AUC, which measures the fraction of assembled transcripts that are in the reference set of transcripts (true positives) and the fraction that are not (false positives) on a range of confidence thresholds (details in Section S1 in Supplementary Data)

In the case of transcript assembly, the ground truth set (the reference transcriptome) is much larger than we will ever see in any single sample since it contains most of transcripts that have been identified and verified. Therefore, the measured sensitivity of any transcript assembly will be very low, in most cases below 0.1%. This means that the AUC value will also be very low. Because there are close to 200,000 transcripts in the reference, even small differences in sensitivity represent a large difference in the true number of transcripts recovered correctly. Because AUC is so small throughout this work multiply the value by $10^{4}$.

### 2.2. Advisor set

Finding an advisor set is especially challenging because Scallop has 18 tunable parameters compared with approximately 5 for MSA, the previous application of parameter advising (see Section S2 in Supplementary Data for a brief description of the Scallop parameters). This means the previously developed method of enumerating a parameter vector universe and then using combinatorial optimization to find an advisor set are infeasible.

### 2.3. Finding an advisor set using coordinate ascent

Iterative optimization strategies such as gradient ascent (Cauchy, 1847), simulated annealing (Kirkpatrick et al., 1983), and coordinate ascent (Zangwill, 1969, sec. 5.4.3) find optimal parameter values by systematically searching high-dimensional spaces based on a specific optimization criteria. For these methods to work well, the parameter landscape should be free from a large number of local maxima as well as large discontinuities. When we examined the parameter behavior for Scallop, we found very few local maxima in the high-dimensional parameter space, which means iterative optimization procedures are unlikely to return a parameter vector that is a poor local maxima (see Section S3 and Fig. S1 in Supplementary Data). We therefore use a greedy coordinate-ascent-based procedure that starts at the default parameter vector to find vectors for our advisor set (see Section S4 in Supplementary Data).

Coordinate ascent will find parameter vectors with higher AUC for an input, but the procedure is slow, and so, it is not viable for finding input-specific parameter choices in practice. Instead, we use coordinate ascent to find higher AUC parameter vectors for a set of examples, and then use the collection of optimal parameters as the advisor set. Since the advisor sets are computed in advance, so as long as the set of examples we use is diverse, meaning it represents the range of possible inputs, the advisor sets can be reused for any new input.

### 2.4. Data

We use three sets of human RNA-Seq experiments to train and validate parameter advising (see Section S5 in Supplementary Data for details):

- ENCODE10 contains a collection of 10 RNA-Seq experiments from the ENCODE database (The ENCODE Project Consortium, 2012) aligned with three alignment tools to produce 30 examples used for training.
- ENCODE65 contains a collection of 65 RNA-Seq experiments, also from ENCODE, produced using a variety of alignment methods.
- SRA contains a collection of 1595 RNA-Seq experiments from the Sequence Read Archive (Leinonen et al., 2010) that have been filtered for quality and aligned using STAR to GRCh38.

## 3. Validating the Transcript Assembly Parameter Advisor

### 3.1. Finding a Scallop advisor set

The ENCODE10 data set is reasonable for training because it is highly diverse and is expected to produce parameter vectors that should generalize. It contains samples that are widely accepted as benchmarks and has examples that have been generated using a collection of commonly used aligners. The coordinate ascent procedure was used to find improved parameters for each sample (see Section S6 and Fig. S2 in Supplementary Data).

Most of the parameters' values across the 30 training samples are quite different, meaning there is unlikely to be one parameter choice that works well for all of the training examples (see Table S1 in Supplementary Data). Even so, given the output of the optimization, a new default parameter setting could be recommended for some single parameters. This is the case for “minimum mapping quality” where almost all samples used a value of 11 rather than the default of 1, which means better AUC can be achieved by disregarding imperfectly mapping or multimapped reads (note these quality values are specific to the aligners we have chosen). In addition, we found that for most training examples, the “minimum transcript length increase” found improvement by selecting values that are much smaller than the default, which can be interpreted to mean that if smaller exons are allowed, the AUC can be improved. In fact, we find that the parameter vector found for SRR545723/TopHat2 had a higher AUC for all of the samples in the ENCODE10 and would be the best vector to use as the default.

### 3.2. Assessing the generality of learned parameter vectors

The ENCODE65 and SRA data sets show the performance of advising in two scenarios: ENCODE65 is used to show that on a large number of samples from a range of aligners (possibly using nondefault parameter settings), advising for Scallop provides a higher AUC transcriptome. SRA contains a large number of samples that have all been preprocessed in the same way with respect to the aligner and gives some insight into the improvement that can be gained in a high-throughput environment.

**FIG. 3.**
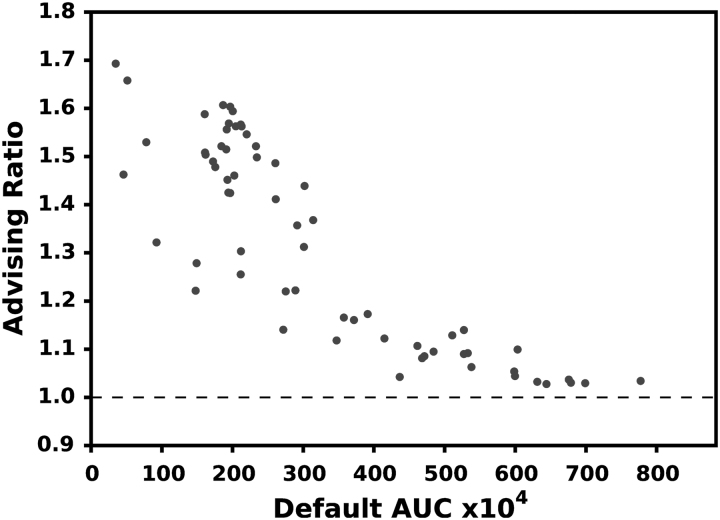
AUC improvement for ENCODE65. Each point is a single experiment positioned by the AUC of the default Scallop parameters (horizontal axis) and the ratio of the advised AUC over the default (vertical). A value above 1.0 indicates an improvement.

**FIG. 4.**
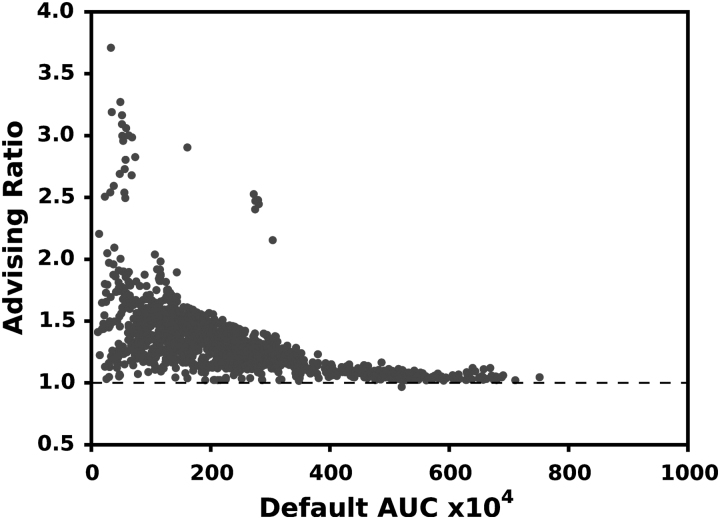
AUC improvement for the SRA assemblies. Each point is a single experiment positioned by the AUC of the default Scallop parameters (horizontal axis) and the ratio of the advised AUC over the default (vertical). A value above 1.0 indicates an improvement. For this test, the default was excluded from the advising set, but it can be included in practice to ensure that the AUC is never reduced.

Figures 3 and 4 show the relative increase in AUC for all the 65 examples in ENCODE65 and the 1595 samples in SRA, respectively. Using advising increases the AUC of predicted transcriptomes by a median of 31.2% in ENCODE65 and 28.9% for SRA. When the default parameter vector performs well, there is a smaller increase in AUC. This is reasonable since there is less room for improvement for these samples.

It may be the case that the available resources may not support using all 31 parameter vectors found using coordinate ascent without a loss in wall clock time. For that case, we developed smaller subsets using methods first described in DeBlasio and Kececioglu (2017a), shown in Table S2 in the Supplementary Data. These smaller advisor sets can then be used in resource-limited environments (see Section S6.2, Tables S3 and S4, in Supplementary Data). We see improvements of 18.2% and 25.6% using as few as two parameter vectors for ENCODE65 and SRA data, respectively.

To confirm that the increase in accuracy is due to our advisor set construction method and not an artifact of having multiple choices of parameter vectors, a collection of random parameter vectors was generated and used for parameter advising (see Section S7 in Supplementary Data). On some examples, the performance is similar between the constructed and random advisor sets (Fig. S3 in Supplementary Data) but the average increase in AUC is much higher for the coordinate ascent advisor set (median AUC increase of 31.23 versus 5.59). In all of the examples from ENCODE65, the coordinate ascent sets outperform the random ones.

In Section S8 in Supplementary Data, we have shown some examples of the running time of using our advisor for Scallop.

### 3.3. Advising for StringTie

To show the generalizability of this method, we also applied it to the StringTie transcript assembler. As before, we ran coordinate ascent on the 10 experiments in ENCODE10, now using StringTie, to select the 30 nondefault parameter vectors (shown in Supplementary Table S5). Since StringTie has only nine tunable parameters, the coordinate ascent time was much shorter, but the increase in accuracy was still observed. For the 30 coordinate ascent runs, we saw a median increase in AUC of 12.2% on ENCODE10.

**FIG. 5.**
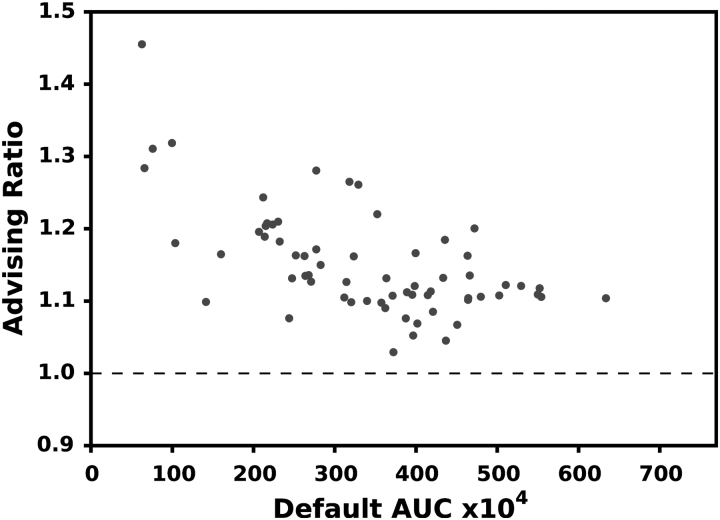
AUC improvement for the ENCODE65 assemblies using StringTie. Each point is a single experiment positioned by the AUC of the default StringTie parameters (horizontal axis) and the ratio of the advised AUC over the default (vertical). A value above 1.0 indicates an improvement.

Figure 5 shows the advising ratio for the 65 RNA-Seq samples from ENCODE65. For these examples, the median gain in AUC is 13.1% over using only the default parameter vectors. For the StringTie assembler, samples with lower AUC using the default parameter vectors still generally have higher advising ratios, but this correlation is not as strong as with Scallop.

### 3.4. Justification for a reference-based advising metric

Comparison with the reference transcriptome is the commonly used metric to benchmark reference-based assemblers. However, the performance of AUC is bounded by the completeness of the reference transcriptome. By definition, all transcripts in an assembly that do not map to the reference provide a reduction of AUC, even those that represent true novel transcripts. Here, a novel transcript is one that is present in the sample but has not been included in the reference yet.

We tested the ability to improve transcript assembly parameter choices of three alternatives to AUC, which do not rely on the reference transcriptome in simulation where we know the ground truth (see Section S9 and Fig. S4 in Supplementary Data). The metrics we compare with are Transrate (Smith-Unna et al., 2016), “number of reads” mapped to the transcriptome using Salmon, and a linear combination of the previous two metrics and other features.

The transcript assemblies constructed using the parameter vectors that optimized using AUC as the objective recover more of the ground truth than those constructed using the default parameter vector. For the other metrics, the transcript assemblies constructed with the parameter vectors found though optimization often showed a decrease in accuracy with respect to the transcripts constructed using the default parameter vector. These metrics are being led astray by not incorporating the knowledge contained in the reference transcriptome.

## 4. Conclusions

Our results show that sample-specific parameter vectors are important for developing any genomic pipeline that includes transcriptome assembly as a step. We begin to answer the question of how to produce transcriptome assemblies effectively for any input without sacrificing quality or expanding manpower. This is done using a combination of parameter tuning through exploration using coordinate ascent and the established method of parameter advising. Two key insights that made this merger viable and distinguish transcriptome assembly from other domains are: (1) the parameter landscape likely having few local maxima, which means coordinate ascent rather than exhaustive enumeration can be used to find advisor sets, and (2) that a small, but representative, number of training examples are sufficient to provide a large increase in AUC over the default parameter setting.

The coordinate ascent procedure is a useful means for finding parameter settings that increase the AUC of predicted transcriptomes. The best results can generally be achieved by simply following this procedure for each new input, although at a high running time. Our implementation of coordinate ascent does not allow steps to be taken in multiple directions at once. It is thus difficult to efficiently parallelize this process. In other words, coordinate ascent finds more accurate parameter vectors at the cost of large computational time. Instead, we have developed a method, which can be reapplied to any domain that has the same parameter behavior we observed, to find an advising set that is as diverse as the training examples used.

One drawback of using a single example to find each parameter vector in the set is that coordinate ascent is likely to overfit to the training examples. We have shown that even with this potential issue, we are able to improve the quality of the transcripts produced according to the AUC measure.

All of the results that we have shown assume that all transcripts in a sequencing sample can be assembled with a single choice of parameter vector. While this is an assumption made by most transcript assemblers, it may not be true in practice. One extension of this work is to provide *transcript-level* parameter choices to help improve the assembly quality. This would require the adaptation of AUC, or some other metric that could work well for parameter optimization, to provide transcript-level assessment.

The method we have described automates the task of parameter selection for Scallop and StringTie, and improves the quality of produced transcriptomes according to the area under the curve measure comparing the output to the reference transcriptome database. As we have shown, this measure works better for the task of parameter optimization than the existing de novo metric. However, our results also show that there is room to improve this measure since the optimal parameter vector recovers more “unknown” transcripts than those optimized for any of the metrics we tested. Unknown transcripts can sometimes be the most interesting, and this measure of accuracy penalizes novelty by definition.

## Supplementary Material

Supplemental data

## Availability

Parameter advisors for both Scallop and StringTie are available on Github at https://github.com/Kingsford-Group/scallopadvising
