# Supplementary material for: More Accurate Transcript Assembly via Parameter Advising
Source: J Comput Biol. 2020 Aug 4;27(8):1181–9. doi: 10.1089/cmb.2019.0286 (PMC7415876; doi:10.1089/cmb.2019.0286)
Supplement: Supplemental data [file Supp_Data.pdf]

Supplemental material for
"More accurate transcript assembly via parameter advising"

Dan DeBlasio*, Kwanho Kim and Carl Kingsford

S1 Calculating AUC

We calculate the AUC of a transcript assembly by allowing `Scallop` to output all predicted transcripts regardless of their minimum coverage. The minimum coverage of a transcript is the minimum number of reads that are aligned to each position along the transcript's length and is typically used to filter transcripts used for analysis. We allow the tools `GFFCompare`[1] and `GTFCuff`[2] to calculate an AUC by finding the precision and recall of the transcripts at various cutoffs of this value.

Because the reference transcriptome contains a large number of transcripts the computed sensitivity is very low, and in turn so is AUC. Since we may not know how many transcripts to expect in a sample ahead of time we cannot use any type of numerical correction. While the values are small, comparing them will still indicate relative performance improvements with respect to number of true and false positives using various parameter choices. In this work, as opposed to Shao and Kingsford (2017), we choose to not separately evaluate multi-exon transcripts and single-exon transcripts but rather maximize a combined AUC.

S2 Scallop Parameters

The `Scallop` transcript assembler generates a transcriptome from a set of reads that have been aligned to a reference genome. It first splits the genome into regions of non-overlapping reads, which are called bundles. These bundles can be thought of as genes or groups of overlapping genes. Then, within each bundle a splice graph is constructed based on the split reads that define possible exon boundaries. Paths through the splice graph define potential transcripts, and the final set of transcripts is formed by decomposing the splice graphs into paths while trying to respect as many of the read mappings as possible. The tunable parameters of `Scallop` govern various stages of this process, but we treat the application as a black box and do not examine the actual function of each parameter only how the manipulation of it's value impacts transcript quality.

S3 Analyzing Parameter Behavior

To determine the relationship between AUC and the value of the `Scallop` parameters, we calculate the AUC of the assemblies produced when varying a single parameter's value while keeping the remaining parameters at their default. Figure S1a shows the effect of varying the "minimum subregion gap" and "minimum transcript length, base" parameters. Figure S1b shows the relationship between the parameters and AUC when varying both at the same time. Note that throughout this

[1]https://github.com/gpertea/gffcompare
[2]https://github.com/Kingsford-Group/rnaseqtools

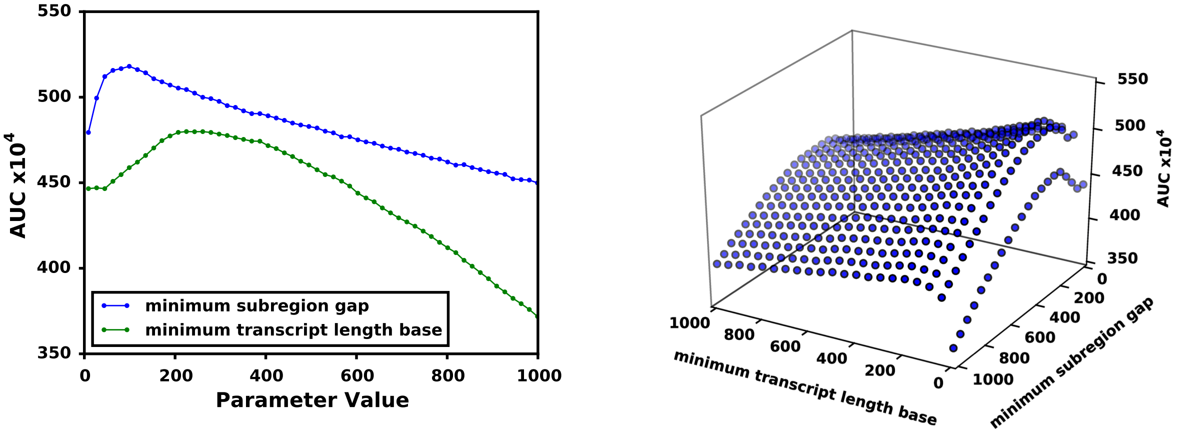

(a) Single Parameter Behaviour (b) Paired Parameter Behaviour

Figure S1: **AUC for various values of the "minimum subregion gap" and "minimum transcript length base" parameters.** The points in the plot show the area under the curve (vertical axis) for the transcriptome produced by changing the value each of the parameters either (a) alone or (b) together leaving all other parameters their default values on SRR534291/`HISAT` from `ENCODE10`.

work, we multiply area under the curve values by 10^4 for ease of presentation. AUC is a value in the range $[0, 1]$, but generally for transcript assembly the value is very small, typically < 0.1.

We examined the parameter behavior curves for several experiments from the ENCODE database, and found that the curves for all 16 continuous parameters and all of the pairs of parameters tested contained only one large visible local maximum. These tests suggest that there may be very few local maxima in the high-dimensional parameter space which means iterative optimization procedures are less likely to get stuck at poor local maxima.

S4 Coordinate Ascent Procedure

One dimension (parameter) at a time, we examine the AUC of the parameter vector with that parameter changed by one step in each direction and update our current vector if we see an improvement. We continue tuning one dimension until no more improvements are made. Our procedure is deterministic meaning we would never take a step that decreases (or maintains) AUC, unlike many implementations of coordinate ascent which include some randomness in decision making. The step size for each dimension has a time-versus-granularity tradeoff: the larger the step size, the less time spent in low AUC regions of the landscape; but when the size is too large, the maximum may be repeatedly stepped over without ever being found. Rather than use a computed step size for every coordinate in each iteration like many implementations of coordinate ascent, we use predetermined but decreasing step sizes, taking inspiration from simulated annealing. We start with different step sizes in each dimension that are large relative to the default value for that parameter, and any time we interrogate the whole set of parameters without making any change we decrease all of the step sizes by a factor of $\frac{1}{4}$ and repeat the process. We continue optimizing the parameters in this manner until both: (1) all of the step sizes are small (1 for integer and 0.01 for real valued parameters)

and (2) no more improvements can be made within one step from the current parameter vector. For the tunable parameters in `Scallop` that accept only binary values, we tested both options each time the parameter was explored.

S5 Data

Details about the datasets used are below. All of experimental identifiers and command line arguments are available at: https://github.com/Kingsford-Group/scallopadvising

S5.1 `ENCODE10`

`ENCODE10` contains a collection of 10 RNA-Seq experiments from the ENCODE database that were used to benchmark `Scallop` and have been extensively used to evaluate transcriptome assembly tools. 30 examples were produced by aligning each sample to the human reference genome (GRCh38) using three tools: `HISAT`, `STAR`, and `TopHat2`. A subset of these examples was used to find the `Scallop` default parameter vector in Shao and Kingsford (2017).

S5.2 `ENCODE65`

`ENCODE65` contains a collection of 65 RNA-Seq experiments, also from ENCODE, that were not included in `ENCODE10` and that had preexisting alignments in the database. These alignments are produced using an aligner selected by the group that submitted the sample and are mapped to either GRCh37 or GRCh38.

S5.3 `SRA`

`SRA` contains a collection of 1595 RNA-Seq experiments from the Sequence Read Archive (Leinonen *et al.*, 2010) that have been filtered for quality. We eliminate any sample that contained very few reads ($<$ 1GB sequence file) or aligned aligned ($<$ 1GB alignment file) since this is an indication that the experiment may be degraded in some way. All remaining samples were aligned using `STAR` to GRCh38.

S6 Training Using `ENCODE10`

Table S1 shows the parameter vectors found for each of the 30 examples in `ENCODE10`. While all parameters were tuned when optimizing parameter choices, for some parameters the final parameter vectors never included non-default parameter values, namely “maximum dynamic programming table size”, “maximum edit distance”, and “minimum router count.” When training we placed no restrictions on any of the ranges of values any particular parameter can take on, so while the values may seem somewhat unintuitive to domain experts, they are the values that give the highest AUC value for the training example. The deviation of the parameter vectors from the default is not surprising given that in this work we are optimizing AUC on all transcripts, rather than only multi-exon transcripts as was done previously.

S6.1 Advising on the training set

Figure S2 shows the AUC for all of the samples from `ENCODE10`. For each example (a sample combined with an aligner) there are two values shown: the AUC of the parameter vector produced

Table S1: Optimal parameter vectors found by coordinate ascent

ID	Experiment	Aligner	ICC	NE	BG	EL	FL	MQ	NH	SBH	SG	SL	SO	TLB	TLI	UM	US	1	2	4	8
1	SRR545723	`TopHat2`	2.00	1000	1359	20	3	11	20	1	39	11	1.50	454	8	false	false	X		X	X
2	SRR307911	`TopHat2`	2.00	1000	1032	20	2	11	21	1	52	15	1.50	432	19	false	false				
3	SRR534291	`TopHat2`	0.75	51	785	25	3	1	9	1	121	16	1.51	503	8	false	false				X
4	SRR387661	`TopHat2`	0.22	1000	999	20	3	11	20	1	53	15	1.50	429	19	false	false		X		
5	SRR315334	`TopHat2`	1.50	1000	1425	20	3	11	20	1	50	16	1.50	528	1	false	false				
6	SRR307903	`TopHat2`	1.61	250	733	2	0	11	2	1	23	26	0.75	521	2	false	false				
7	SRR545695	`TopHat2`	2.00	1000	852	20	2	2	104	1	5	7	1.50	432	19	false	false				
8	SRR534319	`TopHat2`	2.27	1000	306	20	0	1	20	1	7	11	1.50	346	21	false	false				
9	SRR315323	`TopHat2`	0.22	205	945	20	0	2	20	1	39	15	1.16	409	8	true	true				
10	SRR534307	`TopHat2`	2.22	1000	78	32	3	11	16	2	0	32	1.50	512	8	false	false		X		
11	SRR545723	`HISAT`	1.75	11000	628	20	3	11	20	2	8	7	1.50	408	8	false	false				
12	SRR307911	`HISAT`	99.66	1000	1828	3	5	11	3	1	43	15	0.17	392	8	false	false				
13	SRR534291	`HISAT`	1.00	11000	851	22	5	11	3	1	120	3	0.17	267	30	false	false				
14	SRR387661	`HISAT`	1.05	454	130	26	4	11	38	1	23	0	0.17	880	0	false	false				X
15	SRR315334	`HISAT`	0.00	282	870	20	4	11	1	1	48	8	1.28	307	21	false	false				
16	SRR307903	`HISAT`	1.42	168	745	9	5	11	2	1	23	6	0.17	396	0	false	false				
17	SRR545695	`HISAT`	5.56	1000	778	26	2	11	25	2	0	0	1.50	360	8	false	false				X
18	SRR534319	`HISAT`	2.00	1000	1669	20	0	11	81	1	4	7	1.50	150	73	false	false				
19	SRR315323	`HISAT`	2.00	685	782	15	0	11	14	1	40	7	1.50	180	50	false	false				
20	SRR534307	`HISAT`	2.00	146	309	41	8	11	10	3	2	0	1.17	370	4	false	false			X	X
21	SRR545723	`STAR`	2.00	11000	455	22	6	11	3	1	3	21	1.26	252	16	false	false				
22	SRR307911	`STAR`	0.22	1000	490	20	3	11	8	1	53	26	0.50	289	24	false	false			X	
23	SRR534291	`STAR`	0.22	11000	481	24	0	2	11	1	123	26	0.24	352	8	false	false				
24	SRR387661	`STAR`	2.00	1000	734	20	4	11	35	2	42	29	1.50	411	1	false	false				
25	SRR315334	`STAR`	0.50	1000	1070	8	0	11	8	1	41	29	0.75	267	33	false	false				X
26	SRR307903	`STAR`	2.00	1000	976	8	5	11	20	1	35	35	0.17	433	8	false	false				
27	SRR545695	`STAR`	2.00	1000	388	20	8	11	89	2	20	23	1.50	307	19	false	false				
28	SRR534319	`STAR`	2.00	800	1574	17	4	2	20	1	8	18	1.50	307	4	false	false				X
29	SRR315323	`STAR`	2.00	11000	1043	20	3	7	20	1	33	35	1.50	150	50	true	true				
30	SRR534307	`STAR`	1.58	292	54	20	0	11	9	3	0	29	1.50	320	8	false	false			X	X
	Default		2.00	1,000	50	20	3	1	20	1	3	15	1.50	150	50	false	false				
	Initial step size		10.00	10,000	100	100	10	10	100	10	10	20	10.00	500	100	n/a	n/a				

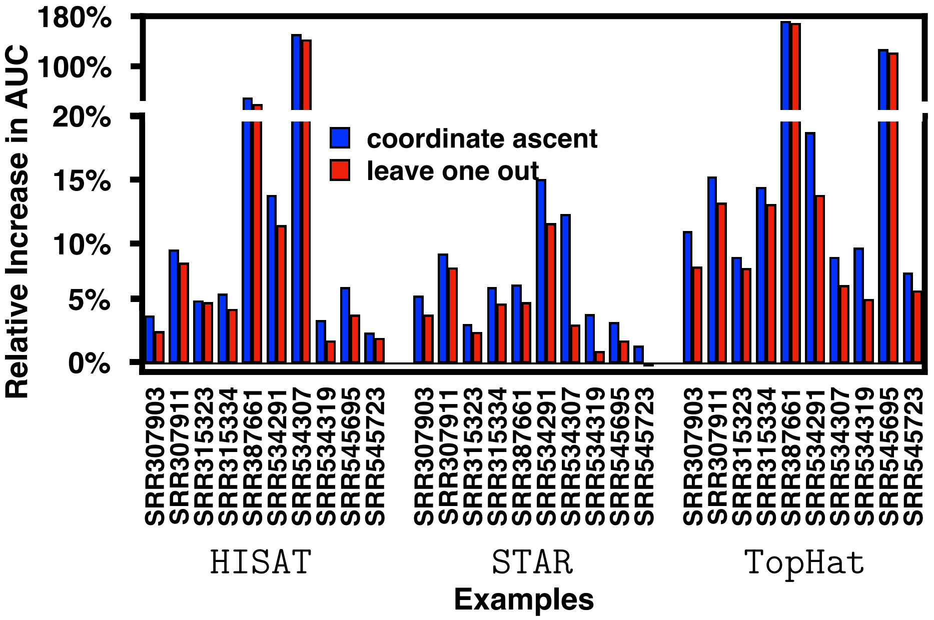

Figure S2: **Increase in AUC for examples in ENCODE10.** Each of the 30 examples is listed on the horizontal axis. Each bar's height is the normalized difference between the default AUC and that of the assembly produced using either coordinate ascent (blue) or leave-one-out (red).

as a result of coordinate ascent, and the AUC of the leave-one-out advising parameter vector. For the leave-one-out experiment, advising was limited to the 18 parameter vectors that were learned on examples produced using the 2 aligners and 9 samples that are different from the example being tested. This test shows that the parameter vectors learned on specific examples, can generalize and improve the AUC for other unrelated examples.

S6.2 Parameter vector subsets

In reduced resource environments (e.g., when 31 processors are not available), it may be desirable to consider fewer parameter settings to keep the number of parallel processes smaller than the number of available threads. We used the oracle set-finding method described by DeBlasio and Kececioglu (2017b,a) to find a subset of parameter vectors that maximizes the average AUC for advising.

Advisor subsets are found using an integer linear program that has two sets of binary variables: one variable for each parameter vector, and one for each example/parameter pair. Where an example/parameter pair is a parameter vector used to assemble an example. Constraints are used to ensure that only one pair for each example is chosen and that the associated parameter setting is also chosen. The objective is then to maximize the sum of the accuracies of the chosen pairs while only selecting a predefined number of parameter vectors. Using the samples in `ENCODE10`, we found advising subsets of 1, 2, 4, and 8 parameter vectors. The advising subset choices are shown Table S2, here only the parameter vectors are shown that contribute to one of the subsets. Each column of the table shows one of the 4 limited sets, and parameter vectors (rows) are in that set if the cell contains an "X". An advising set of size 1 is equivalent to finding a new default parameter vector since it maximizes the average accuracy across the training examples.

Table S2: Parameter vector subsets

Experiment/Aligner	Subset size 1	2	4	8
SRR545723/`TopHat2`	X		X	X
SRR534291/`TopHat2`				X
SRR387661/`TopHat2`		X		
SRR534307/`TopHat2`		X		
SRR387661/`HISAT`				X
SRR545695/`HISAT`				X
SRR534307/`HISAT`			X	X
SRR307911/`STAR`			X	
SRR315334/`STAR`				X
SRR534319/`STAR`				X
SRR534307/`STAR`			X	X

S6.2.1 `ENCODE65`

When using the resource limited sets on the samples in `ENCODE65`, the median increase remains 18.2%, 19.0% and 24.4% for sets of 2, 4, and 8 parameter settings respectively. Even with these small sets, there is a large increase in AUC.

Table S3 shows the percentage of examples from `ENCODE65` for which each of the 31 (the whole set of parameter vectors learned on `ENCODE10` plus the original default parameter vector), 8, 4, and 2 parameter vectors provides the maximum AUC. Only 9 of the 31 (29%) parameter vectors provide the maximal AUC for any sample in `ENCODE65`, but a greater fraction (50%) of the parameter vectors are maximal for each of the reduced size advisor sets. The parameter vector learned from SRR387661/`TopHat2` gives the highest AUC on `ENCODE65`, as opposed to the vector SRR545723/`TopHat2` which is optimal on `ENCODE10` (376.8 vs 373.6) which is why it is used most in the vector subset of size 2.

S6.2.2 `SRA`

The resource-limited advisor sets also show an increase in accuracy for examples in `SRA` where the median improvement is 25.6%, 24.1% and 24.3% with 2, 4, and 8 parameter vectors, respectively. The increase in AUC actually goes down slightly when increasing the size from 2 to 4. This means that the parameter vectors and sets may be slightly overfit to the training data. Table S4 shows the percentage of examples from `SRA` for which each of the 31, 8, 4, and 2 parameter vectors provides the maximum AUC. Surprisingly, even though all of the examples are aligned using `STAR`, many of the higher-frequency parameter vectors had been optimized for examples that were aligned using `TopHat2`. Ties, if any existed, would be resolved in alphabetical order of the experiment name then aligner but no ties for the maximum AUC were found in `SRA` or `ENCODE65`.

S7 Random Advisor Sets

To confirm that the increase in accuracy is due to our advisor set construction method and not an artifact of having multiple choices of parameter vectors, a collection of random parameter vectors were generated and used for parameter advising. A range was defined for each tunable parameter by examining all of the values that provided an increase in AUC for any example at any stage in

Table S3: ENCODE65 Parameter Use

Experiment/Aligner	Subset size 31	8	4	2
SRR545723/TopHat2	0	10.8%	38.5%	
SRR534291/TopHat2	3.1%	33.8%		
SRR387661/TopHat2	0			92.3%
SRR534307/TopHat2	0			7.7%
SRR315323/TopHat2	21.5%			
SRR307903/TopHat2	7.7%			
SRR315334/TopHat2	4.6%			
SRR534319/TopHat2	1.5%			
SRR387661/HISAT	7.7%	10.8%		
SRR545695/HISAT	0	0		
SRR534307/HISAT	0	0	0	
SRR534319/HISAT	1.5%			
SRR307911/STAR	0		61.5%	
SRR315334/STAR	4.6%	44.6%		
SRR534307/STAR	0	0	0	
SRR534319/STAR	0	0		
SRR534291/STAR	47.7%			

Note: only parameter vectors in subsets or those that are chosen by an example from ENCODE65 are listed. Values are only listed for parameter vectors in the subset corresponding to a column.

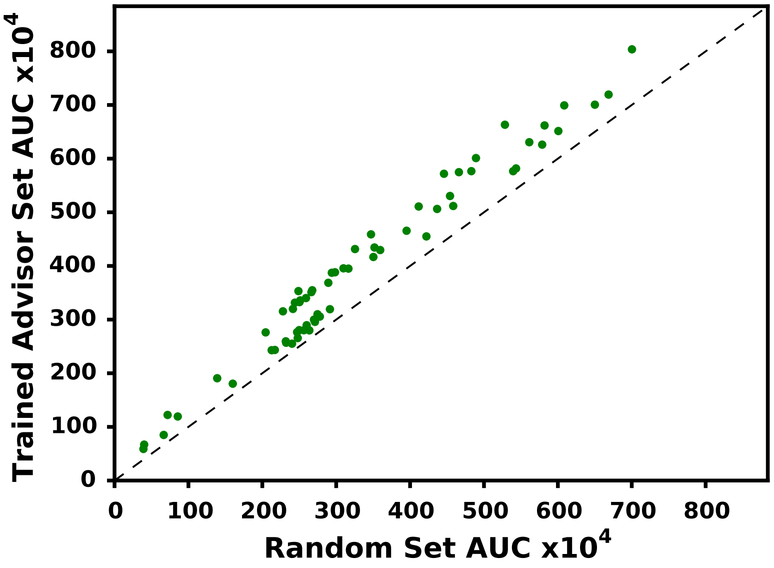

Figure S3: **Comparison to randomly generated advisor sets.** Each point in the chart represents one example from ENCODE65 positioned by the AUC value of using a randomly generated advisor set (horizontal axis) or the trained advisor set (vertical axis) averaged over 100 random replications. Points above the diagonal line show an improvement of training over random.

Table S4: SRA Parameter Use

Experiment/Aligner	Subset ize 31	8	4	2
SRR545723/TopHat2	< 1%	50.9%	67.9%	
SRR534291/TopHat2	3.3%	33.7%		
SRR387661/TopHat2	< 1%			76.5%
SRR534307/TopHat2	4.2%			23.5%
SRR307903/TopHat2	26.9%			
SRR315334/TopHat2	21.6%			
SRR315323/TopHat2	< 1%			
SRR534319/TopHat2	< 1%			
SRR387661/HISAT	< 1%	1.2%		
SRR545695/HISAT	< 1%	< 1%		
SRR534307/HISAT	0	0	< 1%	
SRR534291/HISAT	6%			
SRR315334/HISAT	1.5%			
SRR307911/HISAT	< 1%			
SRR545723/HISAT	< 1%			
SRR307911/STAR	3.2%		2.9%	
SRR315334/STAR	< 1%	7.0%		
SRR534319/STAR	< 1%	5.1%		
SRR534307/STAR	< 1%	2.0%	2.7%	
SRR534291/STAR	19.5%			
SRR307903/STAR	3.7%			
SRR387661/STAR	1.9%			
SRR545695/STAR	1.5%			
SRR545723/STAR	< 1%			
SRR315323/STAR	< 1%			

Note: only parameter vectors in subsets or those that are chosen by an example from SRA are listed. Values are only listed for parameter vectors in the subset corresponding to a column.

coordinate ascent. A random vector was then constructed by selecting parameter values for each parameter uniformly at random in these ranges. In total, 30 such parameter vectors were generated to match the advisor set size developed using coordinate ascent. This randomization procedure was then replicated 100 times to ensure stability of the average.

Figure S3 shows the AUC achieved by parameter advising on `Scallop` using the the coordinate-ascent-derived advising set versus the AUC of advising using the random advisor sets. The default parameter vector was left out of the all advising sets. Because of this, many of the randomly generated advisor sets (29 of 65) led to a decrease in accuracy relative to the default. On some examples the performance is similar between the two sets, but the average increase in AUC is much higher for the coordinate ascent advisor set (median AUC increase of 31.23 versus 5.59). In all of the 65 examples, the coordinate ascent sets outperform the random ones.

S8 Running Time

The wall time of running coordinate ascent is much larger than the running time of any single instance of `Scallop`. For the 30 examples from `ENCODE10`, running coordinate ascent for any single example took between about 40 hours and over 22 days.

Running an advisor would take only as much wall-time as running a single instance of `Scallop` with appropriate resources. Running `Scallop` using the default parameter vector for these same samples takes between $\sim$7 minutes and 1 hour. Even if no parallelization was possible, parameter advising would be able to run in a fraction of the time of running coordinate ascent.

S9 Justification for a Reference-Based Advising Metric

Simulated datasets were constructed using the samples in `ENCODE10`. We restricted the reference transcriptome for each experiment to be the collection of transcripts from the reference that map to assembled transcripts in that experiment. We then limit the set of sequencing reads to those that map to the transcripts in this reduced reference.

Since the reduced reference has all of the transcripts in the sample and nothing else, the AUC using this reduced reference transcriptome in this case is the ground truth accuracy. We can then compare just how well the other metrics are able to recover this known truth set.

From this reduced reference, we sample transcripts to create a smaller "partial reference" that contains approximately 70% of the transcripts. The transcripts were included in the partial reference with probability equal to their frequencies across iterations of coordinate ascent. Transcripts that are observed more often across parameter vectors are more likely to be included. The resulting partial reference closely resembles the actual reference transcriptome in a sense that it contains the majority of frequently encountered transcripts, while missing the ones that are less frequent. To measure and compare the quality of the optimal assemblies under different metrics, we provide only the partial reference in optimization, and check for the improvement by calculating AUC against whole reference.

Metrics that we consider include a de-novo transcriptome assembly quality analysis tool `TransRate`, the number of reads mapped to the assembly using `Salmon` (Num Reads), and linear combination of the `Transrate` feature functions, number of reads mapped, and other features (Linear Sum).

Figure S4 shows the improvement of the parameter vectors found using coordinate ascent when optimizing AUC using the entire restricted reference ("whole AUC") which represents the best achievable optimization, AUC using the subset of restricted reference ("partial AUC") which represents AUC as used in practice, and the 3 metrics above.

Table S5: Optimal parameter vectors for `StringTie` found by coordinate ascent

ID	Experiment	Aligner	M	a	f	g	j	m	t	u
1	SRR307903	`HISAT`	0.13	14	0	52	1	468	false	false
2	SRR307911	`HISAT`	0.11	13	0	54	1	444	false	false
3	SRR315323	`HISAT`	0.10	11	0	37	1	389	false	false
4	SRR315334	`HISAT`	3.03	10	0	58	2	446	false	false
5	SRR387661	`HISAT`	0.93	10	0	0	3	543	false	false
6	SRR534291	`HISAT`	50.92	20	0.01	133	0	483	false	false
7	SRR534307	`HISAT`	3.74	13	0	4	6	474	false	false
8	SRR534319	`HISAT`	0.31	6	0.23	15	3	375	false	true
9	SRR545695	`HISAT`	0.06	10	0.15	11	3	352	false	false
10	SRR545723	`HISAT`	0.11	0	0	11	2	634	false	false
11	SRR307903	`STAR`	0.34	6	0	21	2	464	false	false
12	SRR307911	`STAR`	0.23	11	0.08	71	1	416	false	false
13	SRR315323	`STAR`	50.92	11	0.19	54	0	382	false	false
14	SRR315334	`STAR`	0.57	10	0	43	2	459	false	false
15	SRR387661	`STAR`	50.92	13	0.1	77	4	408	false	false
16	SRR534291	`STAR`	1.01	12	0	58	1	293	false	false
17	SRR534307	`STAR`	50.89	8	0	1	7	596	false	false
18	SRR534319	`STAR`	0.28	9	0.27	39	3	406	false	false
19	SRR545695	`STAR`	0.31	10	0.19	62	4	456	false	false
20	SRR545723	`STAR`	0.46	10	0.01	3	1	551	false	false
21	SRR307903	`TopHat2`	0.06	10	0	64	1	575	false	false
22	SRR307911	`TopHat2`	0.08	8	0	53	1	561	false	false
23	SRR315323	`TopHat2`	0.29	1	0	40	0	536	false	false
24	SRR315334	`TopHat2`	0.07	7	0	38	2	530	false	false
25	SRR387661	`TopHat2`	0.21	6	0	9	3	579	false	false
26	SRR534291	`TopHat2`	12.79	10	0	112	1	492	false	false
27	SRR534307	`TopHat2`	0.12	7	0	2	5	593	false	false
28	SRR534319	`TopHat2`	0.18	5	0.25	39	2	370	false	false
29	SRR545695	`TopHat2`	0.23	8	0.13	14	3	407	false	false
30	SRR545723	`TopHat2`	0.18	3	0	61	1	778	false	false
	Default		0.95	10	0.1	50	1	10	false	false
	Initial Step Size		50	1,000	50	5,000	100	10,000	n/a	n/a

M — fraction of bundle allowed to be covered by multi-hit reads
a — minimum anchor length for junctions
f — minimum isoform fraction
g — gap between read mappings triggering a new bundle
j — minimum junction coverage
m — minimum assembled transcript length
t — disable trimming of predicted transcripts based on coverage
u — no multi-mapping correction

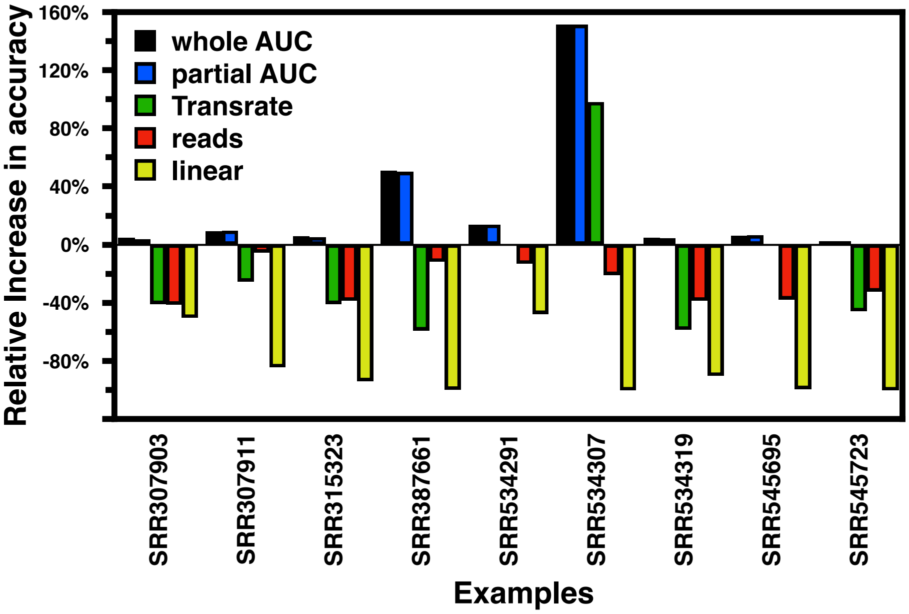

Figure S4: **Relative increase in accuracy over default of using various transcript assembly metrics for coordinate ascent.** The bars represent the difference in accuracy between the optimal parameter choice vector for each metric and the default vector, normalized by the default accuracy for each of the examples from `ENCODE10`.

An ideal test for how much predictive power is lost by optimizing AUC as opposed to other metrics would be to use a fully simulated RNA-seq sample where we know all of the transcripts that are present. We choose to use experiments that more closely resemble biological samples in that they include extraneous sequencing reads that comes from amplification, sequencing, or assembly errors.

An alternate explanation as to why partial AUC finds more accurate parameter vectors is that our accuracy measure is inappropriate and the *de novo*-metric-optimal parameter vectors actually provide more realistic assemblies. if this were true a large number of transcripts assembled using the default parameter vector that map to the reference transcriptome but are actually wrong, since many those transcripts are missing in the *de novo*-metric-optimal assemblies and are replaced with transcripts that do not map to the reference. Even though there is some debate over the completeness of the reference transcriptome (Pertea *et al.*, 2018; Jungreis *et al.*, 2018), it seems unlikely that the number of transcripts that are missing would be large enough to cause such a large discrepancy. In the future, it would be ideal to find some new measure that is some hybrid between AUC and *de novo* assessment that could both use current knowledge but still be able to detect novel transcripts. When this is available, it can replace AUC as the measure for constructing and using advising for transcript assembly.

References

DeBlasio, D. and Kececioglu, J. 2017a. Learning parameter-advising sets for multiple sequence alignment. *IEEE/ACM Transactions on Computational Biology and Bioinformatics* 14, 1028–1041.

DeBlasio, D. and Kececioglu, J. 2017b. *Parameter advising for multiple sequence alignment*, volume 26 of *Computational Biology series*. Springer International Publishing, Cham, Switzerland. ISBN 978-3-319-64917-7.

Jungreis, I., Tress, M. L., Mudge, J., Sisu, C., Hunt, T., Johnson, R., Uszczynska-Ratajczak, B., Lagarde, J., Wright, J., Muir, P., Gerstein, M., Guigo, R., Kellis, M., Frankish, A., Flicek, P., and The GENCODE Consortium 2018. Nearly all new protein-coding predictions in the CHESS database are not protein-coding. *bioRxiv* 360602.

Leinonen, R., Sugawara, H., and Shumway, M. on behalf of the International Nucleotide Sequence Database Collaboration 2010. The sequence read archive. *Nucleic Acids Research* 39, D19–D21.

Pertea, M., Shumate, A., Pertea, G., Varabyou, A., Breitwieser, F. P., Chang, Y.-C., Madugundu, A. K., Pandey, A., and Salzberg, S. L. 2018. CHESS: a new human gene catalog curated from thousands of large-scale RNA sequencing experiments reveals extensive transcriptional noise. *Genome Biology* 19, 208.

Shao, M. and Kingsford, C. 2017. Accurate assembly of transcripts through phase-preserving graph decomposition. *Nature Biotechnology* 35, 1167–1169.
